# Oxidative Stress Impact on the Transcriptome of Differentiating Neuroblastoma Cells: Implication for Psychiatric Disorders

**DOI:** 10.3390/ijms21239182

**Published:** 2020-12-02

**Authors:** Behnaz Khavari, Ebrahim Mahmoudi, Michael P. Geaghan, Murray J. Cairns

**Affiliations:** 1 School of Biomedical Sciences and Pharmacy, University of Newcastle, Callaghan, NSW 2308, Australia; behnaz.khavari@uon.edu.au (B.K.); Ebrahim.mahmoudi@newcastle.edu.au (E.M.); Michael.geaghan@newcastle.edu.au (M.P.G.); 2 Centre for Brain and Mental Health Research, University of Newcastle and the Hunter Medical Research Institute, Newcastle, NSW 2305, Australia

**Keywords:** oxidative stress, SH-SY5Y, neurodevelopmental disorders, psychiatric disorders, neuronal differentiation, immunity

## Abstract

Prenatal environmental exposures that have been shown to induce oxidative stress (OS) during pregnancy, such as smoking and alcohol consumption, are risk factors for the onset of schizophrenia and other neurodevelopmental disorders (NDDs). While the OS role in the etiology of neurodegenerative diseases is well known, its contribution to the genomic dysregulation associated with psychiatric disorders is less well defined. In this study we used the SH-SY5Y cell line and applied RNA-sequencing to explore transcriptomic changes in response to OS before or during neural differentiation. We observed differential expression of many genes, most of which localised to the synapse and were involved in neuronal differentiation. These genes were enriched in schizophrenia-associated signalling pathways, including PI3K/Akt, axon guidance, and signalling by retinoic acid. Interestingly, circulatory system development was affected by both treatments, which is concordant with observations of increased prevalence of cardiovascular disease in patients with NDDs. We also observed a very significant increase in the expression of immunity-related genes, supporting current hypotheses of immune system involvement in psychiatric disorders. While further investigation of this influence in other cell and animal models is warranted, our data suggest that early life exposure to OS has a disruptive influence on neuronal gene expression that may perturb normal differentiation and neurodevelopment, thereby contributing towards overall risk for developing psychiatric diseases.

## 1. Introduction

Psychiatric disorders such as schizophrenia (SZ), autism spectrum disorder (ASD), bipolar disorder (BD), and major depressive disorder (MDD), are characterised by impairments in cognition, communication, behaviour and motor skills that are thought to arise from abnormal brain development, a process that initiates with the differentiation of the neural progenitor cells [1]. While this process extends through late adolescence, and arguably throughout the lifespan of an individual [2], any disturbance can result in the malfunction and maladaption associated with neurodevelopmental disease. Environmental risk factors such as maternal immune activation, maternal stress during pregnancy, prenatal malnutrition and deficiency, and other obstetric complications that result in fetal hypoxia, could also contribute significantly to their development. Lifestyle associated risk factors including exposure to alcohol [3] and cigarette smoking [4,5] during pregnancy are also thought to increase the risk of neurodevelopmental disorders emerging in the offspring. A common adverse cellular event induced by a number of these environmental exposures is oxidative stress [6,7], the condition characterised by the elevation of intracellular reactive oxygen (ROS), such as hydrogen peroxide (H_2_O_2_), as a result of their excessive production and/or deficiency in antioxidant systems [8]. The brain is particularly vulnerable to oxidative stress, due to its high energy requirements combined with high lipid and metal content and relatively low expression of endogenous antioxidant mechanisms [9].

While oxidative stress is thought to be a key factor in the ageing process and age-related pathophysiological conditions, such as neurodegenerative disorders [10,11,12], including Alzheimer’s disease (AD) and Parkinson’s disease (PD) [11,13], it has also been reported in individuals with neurodevelopmental disorders and may in some cases be due to deficiencies in the redox systems. For example, an early proteomics analysis of postmortem samples from SZ patients and healthy individuals reported that nearly half of altered proteins were related to mitochondrial function and oxidative stress responses, and 90% of patients could be distinguished from controls based on differential expression of energy metabolism- and oxidative stress-associated genes [14]. Also, reduced levels or activity of antioxidant enzymes have been observed in patients with SZ [15,16,17], BD [15] and ASD [18]. Interestingly, individuals with first-episode psychosis (FEP) were recently shown to have lower blood levels of endogenous antioxidants in a meta-analysis [19] and also, significantly reduced brain levels of glutathione, the major antioxidant in the brain [20]. This is supported by genetic studies that have revealed associations of redox gene polymorphisms with SZ [17] and other psychiatric disorders [21], reviewed by Koga et al. and Kim et al., respectively. Also, we recently showed that polygenic risk of schizophrenia variants identified by GWAS [22,23] is enriched within two redox-related pathways including NOS1 (Nitric Oxide Synthase 1) and HIF-2 (Hypoxia-Inducible Factor-2) signalling [24]. Although compelling, it remains unclear if the observed alterations in the redox system are contributing to the etiology of neurodevelopmental disorders or are just a consequence or side effect of their pathology [17].

Assuming a causal role for oxidative stress in the development of psychiatric diseases, it is still unclear when these imbalances in the redox system originate and exert their deteriorating effects. While a combination of genetic background and early life [25] or adolescent [26] exposures to oxidative stress have been suggested to induce cognitive impairments and psychotic symptoms, how moderate sub-threshold cytotoxic levels affect CNS development prenatally, remains unknown. We therefore hypothesize that oxidative stress arising from environmental risk factors associated with neurodevelopmental disorders will alter the transcriptional landscape of neurons during differentiation. To test this hypothesis, we used the most widely applied and cited in vitro system for neurodevelopmental and neuropsychiatric studies, the SH-SY5Y neuroblastoma cell line [27]. Despite its tumor origin, this model has several advantages compared to other human neuronal models such as embryonic stem cells, neuronal progenitor cells (NPCs), and induced pluripotent stem cells (iPSCs). These include low cost, feasibility in terms of ease of culture, reproducibility, literature availability, and the unnecessity of ethical approvals which are needed in studies involving primary human neuronal cultures [27,28]. We induced neuroblast differentiation in the presence of oxidative stress and examined gene expression in the mature neuron-like cells, which revealed significant differential expression of hundreds to thousands of genes, mostly involved in the development of nervous and cardiovascular systems, immunity-related processes, and SZ-associated signalling pathways.

## 2. Results

### 2.1. Confirmation of Differentiation

In our co-treatment experiment, SH-SY5Y cells were differentiated to neuron-like cells in the presence of 10μM H_2_O_2_ for seven days and compared to controls treated only with ATRA, whereas in the pre-treatment experiment, cells were exposed to 10 μM H_2_O_2_ for 72 h prior to the addition of ATRA. The cellular morphology appeared identical in both treatments and displayed the typical neurite outgrowth expected for differentiated neurons. This was supported by the expression of neuronal marker genes, including TUBB3, ENO2, MAP2, MAPT, and SV2A for both co-treatment (log2CPM values of 9.6, 7.6, 8, 3.9, and 6.7, respectively) and pre-treatment (log2CPM values of 9, 7, 8.7, 4, and 6.5, respectively) conditions. None of these genes were differentially expressed, except for MAPT (log2FC = −1) in the pre-treatment condition, indicating that the differentiation process was not impaired due to peroxide treatment.

### 2.2. Transcriptomic Response to Oxidative Stress 

**Figure 1:**
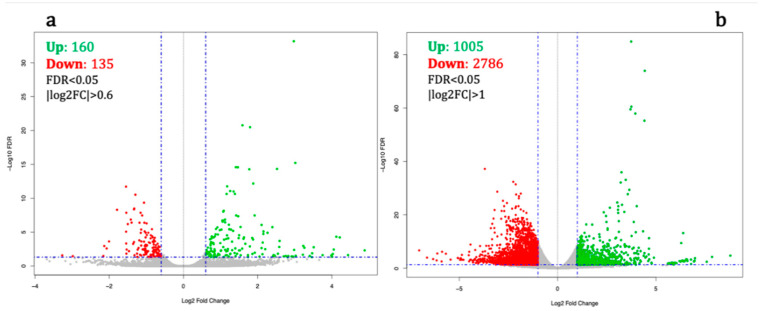
(**a**) Simultaneous treatment of SH-SY5Y neuroblastoma cells with hydrogen peroxide (H_2_O_2_) and ATRA causes up and down-regulation of 160 and 135 genes, respectively, with a false discovery rate < 0.05 and |log_2_FC| > 0.6. In comparison, (**b**) 72 h of treatment with H_2_O_2_ followed by seven days of differentiation with ATRA results in up-regulation of 2786 genes and down-regulation of 1005 genes compared to cells differentiated in the absence of H_2_O_2_. (FDR < 0.05 and |log2FC| > 1). Green and red dots depict up- and down-regulated genes, respectively.

The analysis of normalized cDNA read counts with the edgeR package showed that for co-treatment experiment 295 genes were differentially expressed (FDR < 0.05), of which 160 were up-regulated and 135 were down-regulated compared to cells differentiated in the absence of H_2_O_2_ (Figure 1a and Supplementary Table S1). We also compared the transcriptomic response in SH-SY5Y cells treated with 10M H_2_O_2_ for 72 h prior to the addition of ATRA for seven days. This revealed 3791 genes with statistically significant differential expression (FDR < 0.05), with 1005 and 2786 genes up- and down-regulated, respectively (Figure 1b and Supplementary Table S2). 

**Figure 2:**
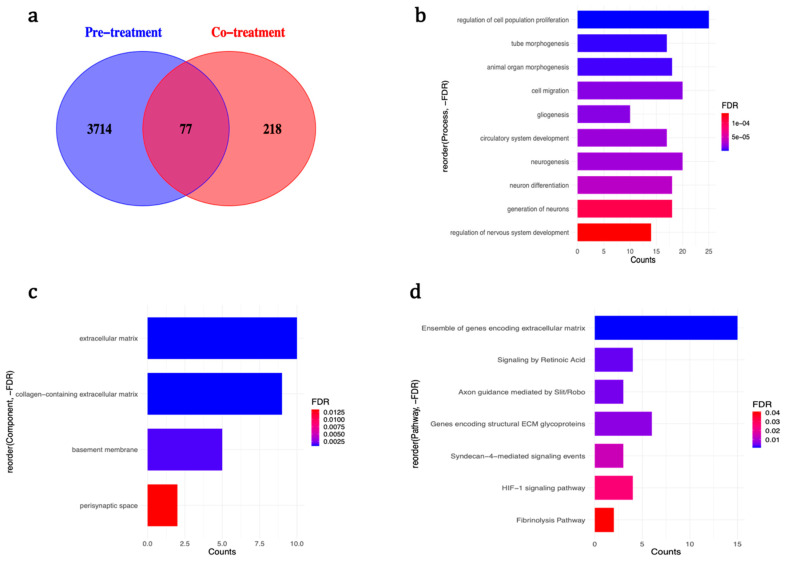
(**a**) Overlap between differentially expressed genes in co-treatment and pre-treatment experiments. The list of common genes is available at Supplementary Table S3. (**b**–**d**): (**b**) the selected enriched biological processes, (**c**) cellular components, and (**d**) signalling pathways, by genes differentially expressed in both experiments. For a complete detailed list, see Supplementary Table S6.

**Figure 3:**
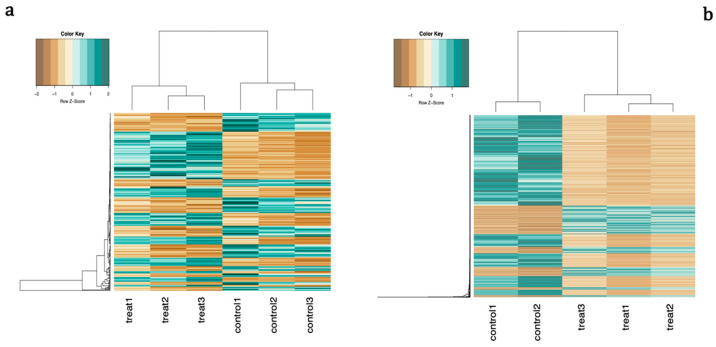
Unsupervised clustering of differentially expressed genes. The dendrogram demonstrates the degree of relatedness of the samples in terms of expression patterns. Each row corresponds to a unique gene, each column shows the expression profile of one sample, and each cell colour reflects the expression level of the corresponding gene in the corresponding sample. Green and brown indicate high and low expression, respectively. Gene expression patterns were clearly distinguishable between peroxide-treated and control samples in cells differentiated either in (**a**) the presence of H_2_O_2_ or (**b**) following 72 h pre-treatment with peroxide.

Surprisingly, only 77 of the differentially expressed genes were common between the two experiments, and all but 6 of them showed the same direction of effect in both conditions (Figure 2a and Supplementary Table S3). Unsupervised hierarchical clustering revealed a clear separation between treatment and control groups for both treatment regimens (Figure 3).

### 2.3. Pathway and Gene-Set Enrichment Analysis

**Figure 4:**
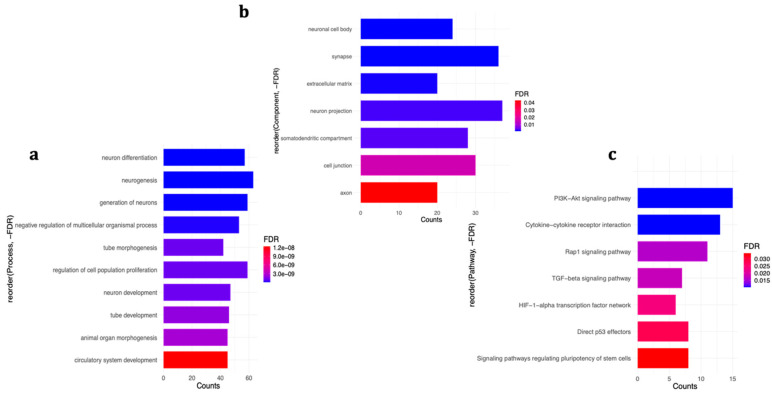
Enrichment analysis of differentially expressed genes in the co-treatment group. (**a**) biological processes, (**b**) cellular components, and (**c**) signalling pathways affected by the differentially expressed genes. For a complete detailed list, see Supplementary Table S4.

The online ToppFun functional enrichment tool was applied to explore the enrichment of differentially expressed genes in biological processes and cellular component gene ontologies, as well as curated signalling pathways. In case of simultaneous treatment with ATRA and H_2_O_2_, the top ranked biological processes were enriched with neurobiologically relevant gene sets including *neurogenesis* (63 genes), *neuron differentiation* (57 genes), and *neuron development* (59 genes) (Figure 4a). The differentially expressed genes were localised in various cellular components, such as *neuronal cell body* (24 genes), *synapse* (36 genes), *extracellular matrix* (20), and *neuron projection* (37 genes) (Figure 4b), and were enriched in pathways including *PI3K/Akt signaling pathway* (15 genes), *Cytokine-cytokine receptor interaction* (13 genes), *TGF-beta signalling pathway* (7 genes), and *HIF-1-alpha transcription factor network* (6 genes), among others (Figure 4c). The complete detailed list of enriched categories and involved genes are presented in Supplementary Table S4. When considered separately, although both up and down-regulated genes were involved in similar biological processes, up-regulated ones were enriched also in the similar cellular components and signalling pathways as the combined analysis, whereas down-regulated genes were only localised in the *cell junction and synapse*, and enriched in *axon guidance* pathway (Supplementary Figure S1). This suggests that the observed enrichments in the co-treatment experiment were mostly influenced by genes with increased expression.

**Figure 5:**
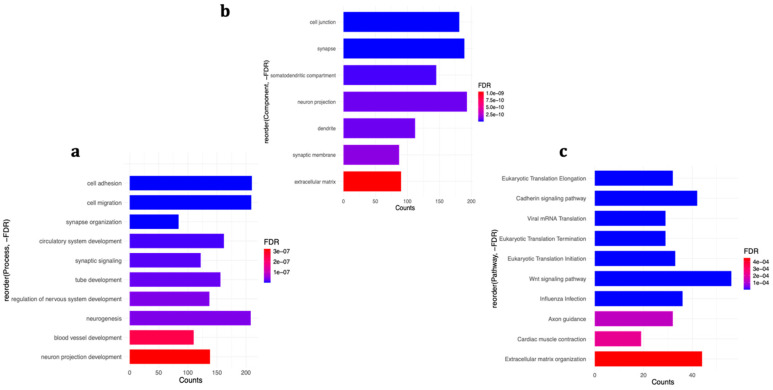
Differentially expressed genes in the pre-treatment group are (**b**) mostly enriched in cell junction and synapse and (**a**) involved in a variety of biological processes and (**c**) signalling pathways. Selected categories are shown. For a complete detailed list, see Supplementary Table S5.

By comparison, when cells were treated with H_2_O_2_ prior to differentiation, the main biological processes affected were related to developmental processes, including *cell adhesion* (210 genes), *synapse organization* (84 genes), *circulatory system development* (162 genes), *tube development* (156 genes), and *regulation of nervous system development* (137 genes) (Figure 5a). The enrichment of differentially expressed genes in various cellular components and signalling pathways is demonstrated in Figure 5b,c, respectively. The complete detailed list of enriched categories and involved genes are shown in Supplementary Table S5. Conversely to the co-treatment experiment, separate submission of dysregulated genes to ToppFun revealed the enrichment of down-regulated genes in similar categories as the differentially expressed genes, especially in terms of biological processes and cellular components, while for up-regulated genes, the results were substantially different, except for some similarity in terms of affected signalling pathways (Supplementary Figure S2), suggesting that the observed enrichments in the pre-treatment experiment were mostly guided by genes with reduced expression.

Gene set enrichment analysis on genes dysregulated in both conditions (*n* = 77) revealed enrichment of the genes in similar biological processes as the co-treatment experiment (Figure 2b), but in rather different cellular components (Figure 2c) and signalling pathways (Figure 2d), compared to either conditions. The complete detailed list of enriched categories and involved genes are presented in Supplementary Table S6.

## 3. Discussion

While the effects of several environmental risk factors for neurodevelopmental disorders are believed to be mediated by the induction of oxidative stress, there is a paucity of knowledge as to how prenatal exposure to oxidative stress contributes to the genomic dysregulation associated with neurodevelopmental disorders. Here, we have used the SH-SY5Y neuroblastoma cell line as a model to explore changes in the transcriptome in response to oxidative stress either before or during neural differentiation. In both conditions we observed significant changes in the transcriptome profile of differentiated cells, with 3791 and 295 differentially expressed genes in cells treated prior and during differentiation, respectively. Surprisingly, there was a substantial difference between cells treated at these different stages, with only 77 genes in common. This suggested that early neural progenitor cells respond very differently compared to those undergoing neuronal differentiation, and therefore the developmental timing of exposure to oxidative stress is likely to be critical to the later onset of psychiatric symptoms.

*Neurogenesis* and *neuron differentiation* were two biological processes enriched in both treatment conditions with 26% of the 77 differentially expressed genes in common involved in these processes. Several of these genes have already been implicated in psychiatric disorders, such as *LIF*, which is located on 22q12.1–q12.2, a particular region known to be a hot spot for schizophrenia, and carries a SNP significantly associated with the disorder [29]. Similarly, *CXCR4* is a highly expressed gene in the central nervous system (CNS) and vital for normal brain morphogenesis during the embryonic stages, and is thought to be a mediator of the pathophysiology of 22q11 deletion syndrome (22q11DS), a chromosome disorder associated with schizophrenia [30]. Deletion of this gene in mice has been shown to be embryonic lethal [31]. Another of these genes, *CD38*, has been implicated in the regulation of astrocytes and oligodendrocytes maturation and differentiation in the developing brain [32], and is also associated with ASD [33], and ASD associated behavioural phenotypes in the knockout mouse model [34]. The pattern receptor gene, *TLR4*, involved in the innate immune system, has been shown to be up-regulated in the PFC of schizophrenia subjects [35], as well as in the blood of individuals with major depressive disorder [36], and peripheral T cells of children with autism [37]. 

Another intriguing observation shared between the two treatments was the enrichment of *signaling by retinoic acid*. Retinoid compounds play a vital role in embryonic development, particularly human brain neurogenesis, and several layers of evidence have implicated the involvement of retinoid signalling dysregulation in the etiology of various psychiatric disorders, such as intellectual disability (ID), ASD, and especially SZ [38]. Four genes associated with retinoid acid signalling were dysregulated in our analyses, including *ALDH1A2*, *PDK1*, *RDH10*, and *AKR1C3*. Our laboratory recently showed that retinoid genes, including *ALDH1A2*, are involved in a SZ-associated polygenic signal [39]. In addition, a study in a Chinese population reported a positive association between *ALDH1A2* SNPs and schizophrenia [40]. *PDK1* is also an interesting gene involved in the PI3K/Akt signaling pathway, which is known to play essential roles during neuronal development, and is related to schizophrenia [41]. Mutation of *PDK1* in mice leads to cognitive impairments and exacerbated disruptive behaviours [42].

Intriguingly, *PI3K/Akt* was the top enriched signalling pathway in our co-treatment condition, with 15 dysregulated genes, including: *PDGFRA*, the expression of which has been found elevated in the prefrontal cortex of schizophrenia patients [43]; *NGFR*, which has essential functions in the developing brain, such as neuronal growth, survival, and differentiation, as well as mediating synaptic and morphological plasticity, and is affected by schizophrenia-associated SNPs [44]; and *FGF14*, which is involved in synaptic plasticity, transmission, and neurogenesis, and suggested as a point of convergence in the pathophysiology of several neurological disorders, including SZ, BD, epilepsy, depression, and addictive behaviours [45].

*Circulatory system development* was another common implicated biological process in our experiments, which might be an interesting observation given that excessive prevalence and premature onset of cardiovascular disease and a higher than expected rate of sudden cardiovascular death have been reported in patients with SZ [46,47], BD [48], ASD [49], and MDD [50]. As an example, cardiovascular disease is estimated to be responsible for a 15 year decrease in life expectancy of SZ patients compared to the general population, and coronary heart disease is the cause of death in more than two-thirds of people with SZ compared to a rate of nearly 50% in the general population [51]. While a significant proportion of this elevated risk is attributable to the use of antipsychotic medications and other confounding lifestyle factors such as higher rates of smoking, poor diet, general neglect of health, and decreased access to health care services [47], our results suggest that oxidative stress insults during critical neurodevelopmental time frames may also contribute to the risk of developing comorbid cardiovascular pathologies. This hypothesis is supported by previous in vivo research showing that the development of cardiovascular diseases in adult offspring of pregnancies with chronic hypoxia is mediated by the induced oxidative stress, and can be prevented by maternal treatment with vitamin C during pregnancy [52]. The role of foetal oxidative stress in the development of cardiovascular diseases has been recently reviewed by Rodríguez-Rodríguez et al. [53].

We also noticed substantial up-regulation of immunity-related genes, up to 400-fold, in the pre-treatment experiment, and the enrichment of the biological process *immune system development* in the co-treatment experiment (FDR = 8.40 × 10^−3^), which might be due to oxidant-induced activation of the redox-sensitive transcription factor NF-kB, that controls the immune and inflammatory responses [54]. In addition, immune-related signalling pathways were affected by some of the differentially expressed genes in both experiments. For example, in the co-treatment condition, *cytokine-cytokine receptor interaction*, which contributes to regulation of immune cell homeostasis and coordination of signal-dependent immune responses [55], particularly inflammation [56], and *TGF-beta signalling pathway*, whose perturbations underlie inflammatory diseases [57], were among the enriched pathways, while in the pre-treatment condition, infection-related pathways, such as *viral mRNA translation* and *influenza infection* were affected. Three of the common dysregulated genes, *TNC*, *CXCR4*, and *FZD7*, were enriched in *Syndecan-4-mediated signalling events*, that are known to be involved in immune responses [58]. *TNC* [59] and *CXCR4* [60,61], in particular, have well-established immunity-related functions. Significantly lower levels of *TNC* protein, which has crucial roles in different aspects of neurodevelopment, were recently observed in the dorsolateral prefrontal cortex (DLPFC) of BD patients with a psychosis history compared to non-psychotic BD subjects [62]. Contrarily, Dominique et al. had previously reported increased expression of this gene in DLPFC of SZ cases in comparison with healthy controls [63].

Several layers of evidence have been suggestive of a relation between the immune system and psychiatric disorders. These include the identification of maternal immune activation as a risk factor for neuropsychiatric illnesses, especially SZ [64]; the increased incidence of immune disorders in individuals with SZ and their relatives [65]; and the observed alterations in concentrations of several pro-inflammatory markers in serum, plasma, cerebrospinal fluid (CSF), and blood cells [66,67,68,69], as well as post-mortem brains [70,71] of schizophrenia patients. There is also an enrichment of genes with disease-associated GWAS common variants in immune signalling pathways in patients with SZ, BD, and MDD [72]; and differential expression (usually up-regulation) of immune genes in the brain and blood of psychiatric patients [73,74,75].

Immunity-related genes with 57 to 400-fold expression increase in our pre-treatment data include *CFI* (Complement Factor I), *CCHCR1*, *IL7R* (also known as IL7RA and CD127), *IKBKE*, *TCIM* (or C8orf4), *IL33* (Interleukin 33), *IL31RA*, and *DPP4*. Of particular interest are *CCHCR1* and *IL33*. *CCHCR1* is located in the major histocompatibility complex (MHC) region, a genetic locus harbouring the most significant common variant association with SZ [22,23]. A common missense allele in this gene was the most significant variant associated with SZ in an exome-sequencing of 2,536 schizophrenia cases and 2,543 controls [76]. *IL33* is a pro-inflammatory cytokine, released postnatally by synapse-associated astrocytes, with a vital role in synaptic pruning. *IL33* deficiency in mice results in overactive brain circuitry, due to too many excitatory synapses [77], suggesting that this gene might be involved in pathological conditions which arise from an imbalance of excitation and inhibition in developing neural systems, such as ASD [78]. Interestingly, dysregulation of *IL33* has been reported in ASD [79], SZ [80], BD [81], recurrent major depressive disorder (rMDD) [82], and perinatal depression [83]. Collectively, our findings suggest that oxidative stress may activate immune-relevant pathways in neurons and potentially exacerbate or potentiate the neurobiological consequences of neuroinflammatory processes, which have been shown to be significant in the pathophysiology of psychiatric disorders [19,84,85].

Consistent with our results, oxidative stress-induced up-regulation of immunity-related genes in neurons has been previously reported in the hippocampal CA1 region of rat pups. With the aim of finding a molecular mechanism for the considerably higher sensitivity of hippocampal CA1 neurons to oxidative stress than CA3 neurons, Wang et al. compared the transcriptome of these cells following exposure to stress. Interestingly, neurogenesis and neuron differentiation processes were affected in both cell types. On the other hand, the observed increased expression of genes related to apoptosis in both regions suggested that other pathways might be responsible for the increased cell death. Gene ontology analysis revealed the enrichment of CA1-specific up-regulated genes in cytokine, chemokine, and inflammatory response, as well as cytokine-cytokine receptor interaction pathway, as the clearest difference between the two hippocampal regions in terms of their responses to oxidative stress [86]; this suggested that immunity-related factors might be the main determinant of cellular response to this type of stress.

**Figure 6:**
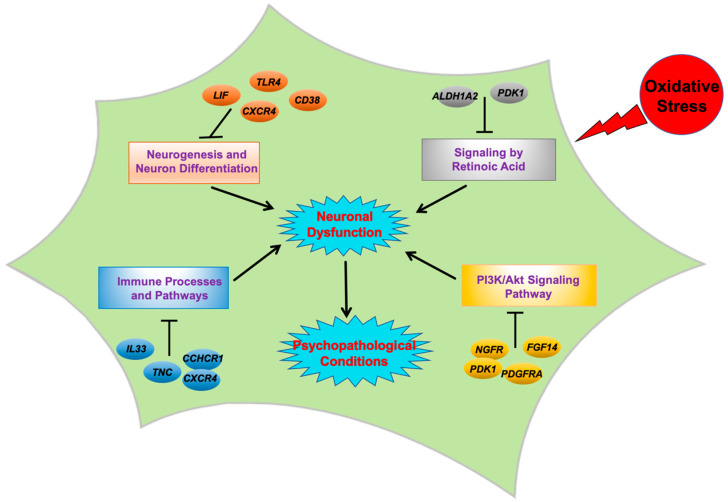
Schematic of the oxidative stress implication in psychiatric disorders. Exposure to oxidative stress results in transcriptomic perturbation in several key pathways that are important for neuronal development and their normal physiology. The resultant dysfunction of neuronal cells may contribute to the risk of initiating and developing psychopathological conditions and/or disorders.

Activation of immune-related pathways in response to oxidative stress, and the resultant neuroinflammation, is well known as a contributing factor to neuronal damage in neurodegenerative disorders [87,88] and Fetal Alcohol Spectrum Disorder (FASD), which is characterized by neurocognitive deficits and behavioural abnormalities [89]. Chronic oxidative stress in the brain was shown to activate CNS innate immunity cells, microglia, to release cytokines and other inflammatory molecules, which are thought to trigger apoptotic death in neurons. In addition, activated microglia also produce ROS, forming a positive feedback loop between oxidative stress and neuroinflammation, that results in progressive damage to the brain [87,90]. Although a similar mechanism has been put forward for developing psychiatric disorders [91,92], the starting point of oxidative stress and inflammatory abnormalities remains unclear [91], since the evidence so far has been based on studies on patients in their early life or adolescence, while the effects of redox imbalance during very early stages of CNS development, such as fetal neural differentiation, remain unknown and therefore, were investigated in this vitro study. Figure 6 represents a schematic summary of the main neurodevelopment- or psychosis-related genes, processes, and pathways affected by exposure to oxidative stress which have been discussed here in detail.

Interestingly, we observed very substantial differential expression of a number of lncRNAs and pseudogenes, especially in the pre-treatment group. Top examples of up-regulated genes include RN7SKP118, RNA5SP195, CTC-534B23.1, KRT18P17, RNU1-67P, RP5-1119A7.11, EEF1A1P7, RP11-606P2.1, RN7SL359P, FAM83C-AS1, RP11-315O6.1, RP1-178F15.4, and CTC-457L16.2, which were increased between 48 and 128-fold compared to control cells. Similarly, RP11-37O16.8, RP3-389A20.5, RN7SL568P, RNU6-1255P, PRELID1P2, and RPS12P10, with 28 to 78-fold expression decrease were the top down-regulated genes. While the functional implications of this is difficult to determine due to the relative lack of understanding surrounding these non-coding genes, it should be noted that long non-coding RNAs have been observed to regulate gene expression at both the transcriptional and post-transcriptional levels, and have been implicated in various psychiatric disorders, such as SZ, ASD, MDD, and ID [93,94]. Similarly, pseudogene products have been recently shown to regulate gene expression [95], and their differential expression has been reported in several psychiatric disorders, including SZ [96]. It is therefore reasonable to speculate that their elevated expression may be a contributing factor to the large number of differentially expressed genes in the pre-treatment condition compared to cells exposed to oxidative stress during differentiation.

In summary, our observations suggest that oxidative stress is a key risk factor in the onset of neurodevelopmental disorders, potentially through the disruption of neurogenesis and differentiation as well as the dysregulation of immune modulating genes in neuronal progenitor cells. While the connection between these two processes is not intuitive, several classes of genes known to be functional in the immune system, such as MHC class I, the complement family, the T cell marker CD3, cytokines, chemokines, and toll-like receptors, display pleiotropy with functions related to neural development, including cell proliferation and differentiation, neurite outgrowth, axonal growth, synaptogenesis, synaptic remodelling and plasticity, and cognition [97]. Oxidative stress is therefore likely to have adverse effects even before the initiation of neuron differentiation, suggesting that the neuronal progenitor cells in the fetal nervous system may be particularly vulnerable in the very early months or even weeks of pregnancy to this exposure, which may therefore significantly contribute to the risk of developing psychiatric disorders later in life. While our study provides evidence, at the transcriptome level, for disruption of neurogenesis upon exposure to oxidative stress, further investigations, such as proteomics analyses, is important to support the observed changes in gene expression at the protein level. In addition, in vivo animal models are necessary in future research to confirm the involvement of prenatal oxidative stress exposure in behavioural changes in the offspring that are thought to be associated with psychiatric disorders.

## 4. Materials and Methods

### 4.1. Cell Culture and Differentiation

The human neuroblastoma SH-SY5Y cells were seeded at a density of 2 × 10^4^ cells per cm^2^ in a cell culture flask, and maintained in Dulbecco’s Modified Eagle’s Medium (DMEM, Irvine, UK, Sigma-Aldrich) cell culture medium at 37 °C in a 5% CO_2_ atmosphere, with the media replaced every 2–3 days. DMEM media was supplemented with 2 mM L-glutamine (Logan, UT, USA, HyClone), 20 mM HEPES (New York, NY, USA, Thermofisher), and 10% foetal bovine serum (FBS, Melbourne, VIC, Australia, Bovogen Biologicals). To accomplish neuronal differentiation, the immature neuroblast cells were seeded into 6-well plates, and after 24 h were treated with 10 μM all-trans retinoic acid (ATRA, Sigma-Aldrich, St. Louis, MO, USA). The cells were incubated while protected from light and the ATRA-supplemented media was refreshed after 72 h. The differentiation protocol was ceased after 7 days of treatment with ATRA, when the cells displayed a neuronal phenotype, with increased growth of neurite projections and stalled cell division.

### 4.2. Application of Oxidative Stress

Oxidative stress was chemically induced in cultured neuroblasts by the addition of 10 μM hydrogen peroxide (H_2_O_2_, Sigma-Aldrich, St. Louis, MO, USA) using two treatment protocols. In the first approach, the cells were simultaneously exposed to H_2_O_2_ and ATRA during the entire 7 days of differentiation (co-treatment protocol). In the alternative pre-treatment approach, cells were treated with 10 μM H_2_O_2_ 72 h before commencement of the ATRA differentiation protocol.

Hydrogen peroxide is widely used for investigating the effects of chronic oxidative stress exposure, due to its high stability compared to other known reactive oxygen species, such as the free radicals superoxide anion and hydroxyl radical, that are too unstable, with a half-life of 10^−10^ to 10^−6^ seconds [98]. The selection of an appropriate, non-cytotoxic concentration of peroxide, that induces oxidative stress without reducing cell viability noticeably, was first based on a literature search for similar studies. Based on the findings, we then treated cells with increasing concentrations of H_2_O_2_, namely 10, 20, 40, 80, and 100 μM, and checked their morphology for 10 successive days through optical microscopy, which revealed that 10 μM H_2_O_2_ had the least adverse effects, and therefore, was chosen for the current study.

### 4.3. RNA Extraction and Analysis of Integrity

Cells were lysed directly in the culture plates by the addition of 1 ml Trizol reagent (Sigma-Aldrich, St. Louis, MO, USA). Lysate was transferred to microcentrifuge tubes and 200 μL of chloroform (Chem-supply, Gillman, SA, Australia) was added before centrifugation at 13,000 g at 4 °C for 10 min in accordance with the manufacturer’s instructions (ThermoFisher). The resultant aqueous phase, containing total RNA, was separated from the organic phase and mixed with 80 μg glycogen (Life Technologies, Mulgrave, VIC, Australia) and 500 μL isopropanol (Chem-supply, Gillman, SA, Australia), and incubated at −20 °C overnight for RNA to precipitate. After centrifuge at 9000 g at 4 °C for 30 min, the supernatant was discarded, and the pellet was washed with 75% cold ethanol twice. Finally, the RNA pellet was re-suspended in nuclease-free water and stored at −80 °C. The concentration and purity of extracted RNA was assessed by the Agilent small RNA kit and the 2100 Bioanalyzer according to the manufacturer’s instructions (Agilent Technologies, Santa Clara, CA, USA). The provided system software was used for automatic calculation of RNA integrity number (RIN). All samples had RIN values above 8.5.

### 4.4. RNA-Seq Library Construction and Sequencing 

Library generation for total RNA-Seq and subsequent sequencing was performed as described before [99]. Briefly, 350 ng of total RNA (RNA integrity number (RIN) ≥ 8.5) was DNAse I treated (1 U/μg RNA, Thermo Scientific, Waltham, MA, USA), before ribosomal RNA depletion using the Ribo-Zero kit (Illumina, San Diego, CA, USA). The Illumina TruSeq Stranded Total RNA Library Prep Kit was applied to construct sequencing libraries according to the manufacturer’s protocol. In order to determine the quality and concentrations of libraries, High Sensitivity DNA Bioanalyzer chip was used, followed by pooling libraries and running in paired-end mode on a NovaSeq 6000 instrument for 150 cycles.

### 4.5. Quality Control, Read Count, and Differential Expression Analysis of RNA-Seq Data

The quality of the sequencing output FASTQ files was assessed using the FastQC software package (v0.11.8), with good-quality reads determined as those without any sequencing adapter content and with a Phred quality score ≥28 for all bases. Following removal of low-quality bases by Cutadapt (v2.10), HISAT2 (v2.1.0) [100] was applied to map the reads to the human genome build hg19. Ultimately, the reads aligning to annotated genes were quantified with htseq-count (v0.7.2) [101].

The R package edgeR (v3.6.1) [102] was applied to perform data normalisation and filtration, and then differential expression analysis was performed, based on the pairwise exact test mode. In order to filter out genes with consistently very low read counts across treatment and control samples, a counts-per-million (CPM) threshold was applied, which represented a minimum of 5 raw counts in the smallest library. Genes with significant differential expression were defined as those with a *p*-value less than 0.05 and a Benjamini-Hochberg FDR less than 0.05. Raw sequencing and processed read-count data are available at the Gene Expression Omnibus (Accession Number: GSE161860).

### 4.6. Pathway and Gene Ontology Analyses

In order to determine the biological processes and signaling pathways that were enriched for the differentially expressed genes, the ToppFun functional enrichment suite, which is an online tool from the ToppGene Suite [103] was used. 

## Supplementary Materials

Supplementary Materials are available online at https://www.mdpi.com/1422-0067/21/23/9182/s1.
