# Supplementary material for: Oxidative Stress Impact on the Transcriptome of Differentiating Neuroblastoma Cells: Implication for Psychiatric Disorders
Source: Int J Mol Sci. 2020 Dec 2;21(23):9182. doi: 10.3390/ijms21239182 (PMC7731408; doi:10.3390/ijms21239182)
Supplement: Supplementary file 1 [file ijms-21-09182-s001.zip › ijms-973390.docx]

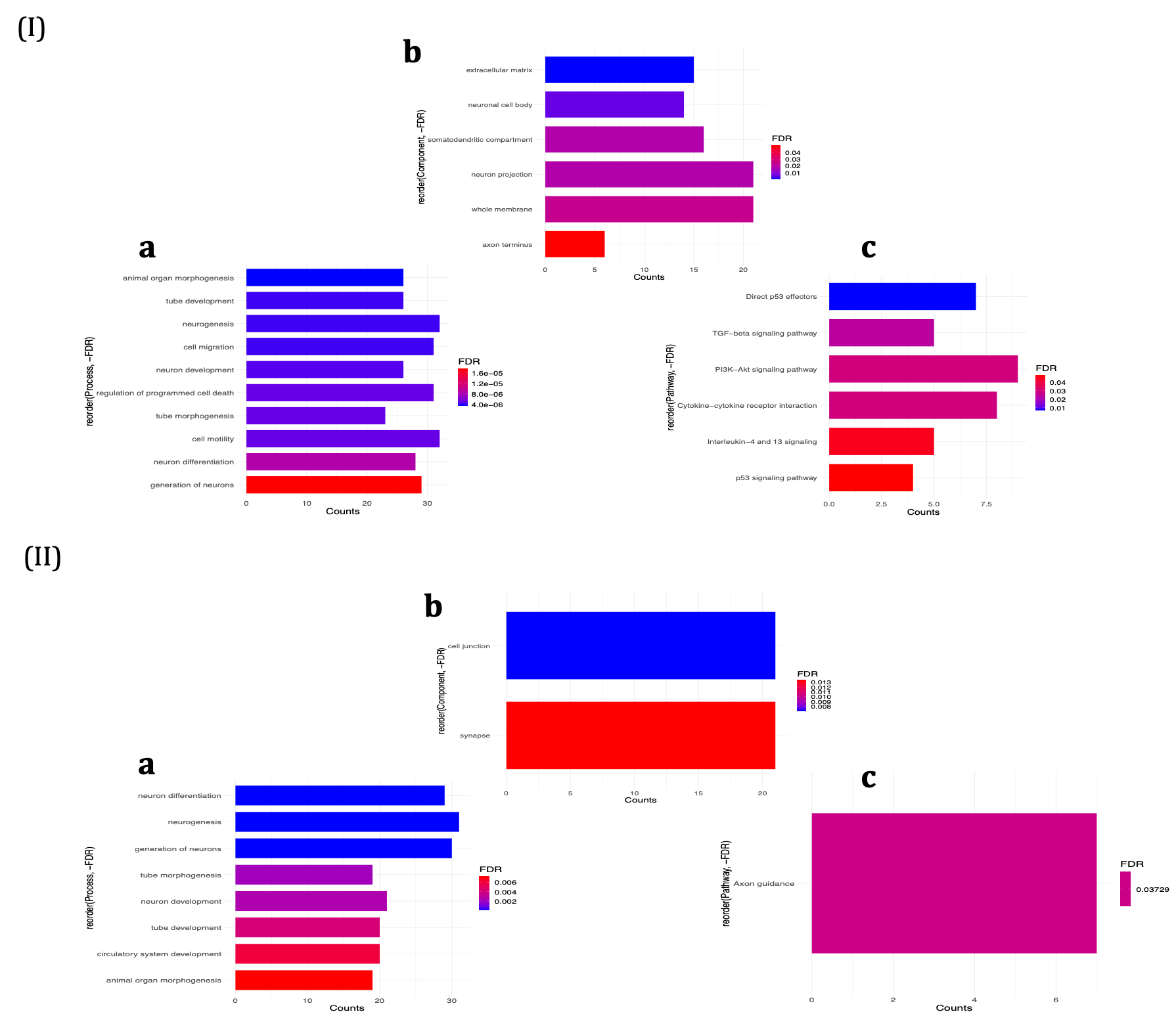


**Figure S1**. Group-specific GSEA on dysregulated genes in the co-treatment condition. (I) Up-regulated genes were enriched in the similar (a) biological processes, (b) cellular components, and (c) signalling pathways as the whole gene set. (II) Although down-regulated genes were involved in the same (a) biological processes as the whole gene set, they were (b) localised in the cell junction and synapse, and (c) enriched in axon guidance pathway only.


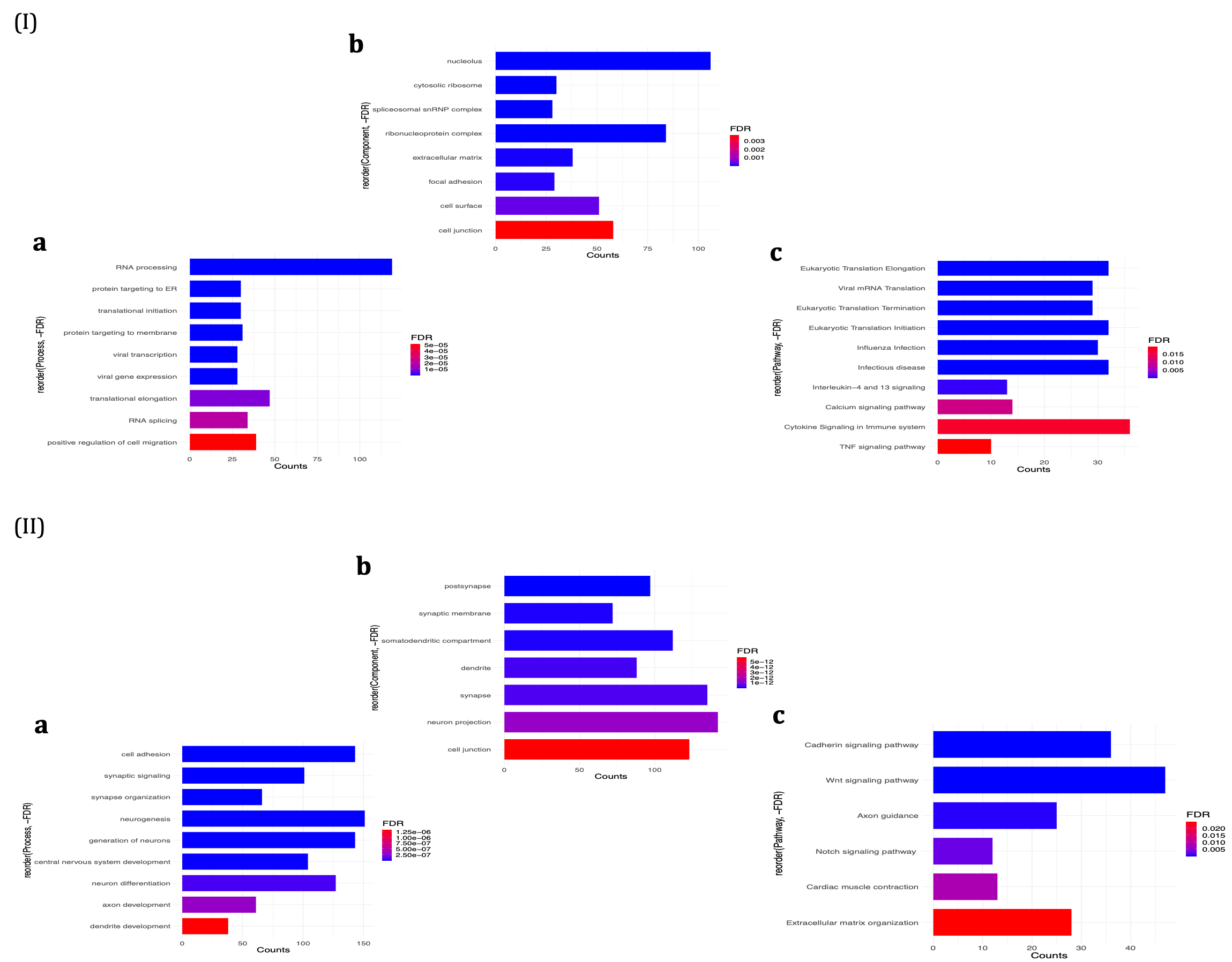


**Figure S2**. Group-specific GSEA on dysregulated genes in the pre-treatment condition. (I) Up-regulated genes shared some similarity with the whole gene set in terms of (c) affected signalling pathways, but were localised and involved in totally different (b) cellular components and (a) biological processes, respectively. (II) Down-regulated genes were enriched in similar categories as the whole gene set (a-c).
